# Author Correction: DNA polymerase epsilon is required for heterochromatin maintenance in *Arabidopsis*

**DOI:** 10.1186/s13059-026-04192-x

**Published:** 2026-07-13

**Authors:** Pierre Bourguet, Leticia López-González, Ángeles Gómez-Zambrano, Thierry Pélissier, Amy Hesketh, Magdalena E. Potok, Marie-Noëlle Pouch-Pélissier, Magali Perez, Olivier Da Ines, David Latrasse, Charles I. White, Steven E. Jacobsen, Moussa Benhamed, Olivier Mathieu

**Affiliations:** 1 Institute of Genetics Reproduction and Development (iGReD), Université Clermont Auvergne, CNRS, Inserm, Clermont-Ferrand, F-63000 France; 2 Present Address: Instituto de Bioquímica Vegetal y Fotosíntesis, CSIC-Cartuja, Avda, Américo Vespucio, 49., Sevilla, 41092 Spain; 3 Department of Molecular, Cell and Developmental Biology, University of California, Los Angeles, Los Angeles, CA 90095 USA; 4 Institute of Plant Sciences Paris-Saclay (IPS2), CNRS, INRA, University Paris-Sud, University of Evry, University Paris-Diderot, Sorbonne Paris-Cite, University of Paris-Saclay, Batiment, 630, Orsay, 91405 France; 5 Howard Hughes Medical Institute, University of California, Los Angeles, Los Angeles, CA 90095 USA

**Author Correction: Genome Biol 21, 283 (2020)**

**https://doi.org/10.1186/s13059-020–02190-1**

Following publication of the original article [1], the authors identified a nomenclature error in the labelling of Fig. S6A. The plant image originally labelled *pol2a-6* should be corrected to *pol2a-12.* During the study, the authors initially referred to the *pol2a* mutant allele *anx2* as *pol2a-6* but renumbered it as *pol2a-12* prior to publication following a careful review of existing nomenclature. A complete list of *pol2a* mutant alleles is provided in Table S1 for further clarification.

The incorrect Fig. S6A is given below.
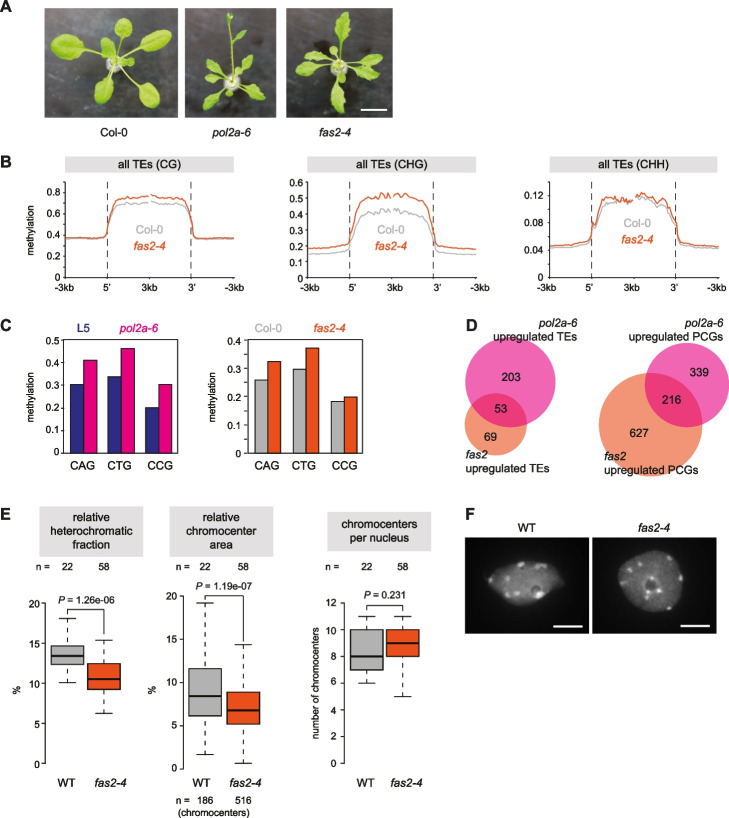


The correct Fig. S6A is given below.
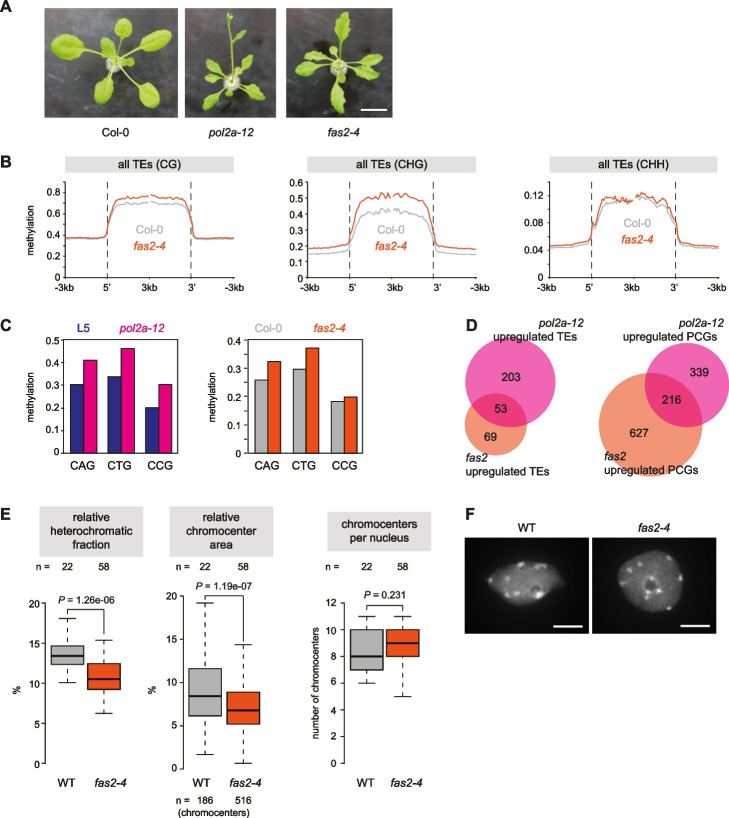


Additionally, the authors wish to clarify that Fig. S6A and Fig. S8D display images of the exact same wild-type (Col-0) and *pol2a-12* plants, which were grown side-by-side in the same experiment. These same control individuals were utilized as standardized baselines for both the *fas2-4* mutant (Fig. S6A) and the *pol2a-12 cmt3-11* double mutant (Fig. S8D). This reuse of control individuals, while experimentally consistent, should have been explicitly stated in the original legends. The corrected figure legends are provided below.

**Figure S6. Comparison of** ***pol2a* and *fas2*** **molecular phenotypes**

**A** Photos of 21-d-old plants. Scale bar: 1cm. **Note that the Col-0 and** ***pol2a-12 images are the same individuals shown in Fig. S8D, as these plants were grown side-by-side and served as common controls for both experiments. B*** Metaplots showing methylation rates in CG, CHG and CHH contexts in *fas2-4* mutants at all TEs (data from Mozgova et al. 2018). Annotations were aligned to their 5’ or 3’ end and average methylation was calculated for each 100-bp bin from 3 kb upstream to 3 kb downstream. **C** Average methylation levels in CHG subcontexts in the indicated samples, excluding positions unmethylated in both the WT and mutant samples in each comparison. **D** Venn diagrams showing the overlap between TEs and PCGs upregulated in *pol2a-12* and *fas2-4*. **E** Relative heterochromatic fraction (left), area of chromocenters normalized to the entire nucleus area (middle) and number of chromocenters per nucleus (right) quantified on DAPI-stained nuclei in WT and *fas2-4*. The number of nuclei analyzed is indicated on top. P-values from an unpaired two-sided Student’s t-test are indicated. **F** DAPI-stained nuclei extracted from WT and *fas2-4* plants. Scale bar: 5 µm.

**Figure S8. Characterization of** ***pol2a cmt3*** **double mutants**

**A** TE methylation changes in CHH context in *pol2a-12* and *pol2a cmt3* normalized to WT and *cmt3*, respectively. Annotations were aligned to their 5’ or 3’ end and average methylation was calculated for each 100-bp bin from 3 kb upstream to 3 kb downstream. **B-C** Transcript accumulation in reads per kilobase per million mapped reads (RPKM) in indicated genotypes. The effect of genotype was verified with a Kruskal–Wallis rank sum test. Significant differences between groups were evaluated by a Dwass-Steel-Crichtlow-Fligner test and are indicated by lowercase letters (*P* < 5e-10 in B, *P* < 5e-2 in C). **D** Representative pictures showing 21-day-old plantlets of the indicated genotypes. Scale bar: 1cm. **Note that the Col-0 and** ***pol2a-12 images are the same individuals shown in Fig. S8D, as these plants were grown side-by-side and served as common controls for both experiments.***

These corrections do not alter the scientific conclusions of the study. The original article [1] has been corrected.

## Supplementary Information

Supplementary file 1: Figure S1. Release of transcriptional silencing in three new *pol2a* mutants. Figure S2. Contribution of H3K27me3 in POL2A-dependent gene silencing. Figure S3. POL2A is required for heterochromatin over-replication in *atxr5/6*. Figure S4. DNA repeats, H3K27me1 and H3K9me2 at *pol2a-12* chromocenters. Figure S5. DNA methylation and H3K9me2 profiles in *pol2a* mutants. Figure S6. Comparison of *pol2a* and *fas2* molecular phenotypes. Figure S7. Changes in small RNA accumulation in *pol2a* mutants. Figure S8. Characterization of *pol2a cmt3* double mutants. Figure S9. DNA methylation profiles in mutant and drug contexts of replicative stress. Figure S10. Comparison of sequencing replicates.
