# Supplementary material for: Author Correction: DNA polymerase epsilon is required for heterochromatin maintenance in Arabidopsis
Source: Genome Biol. 2026 Jul 13;27:224. doi: 10.1186/s13059-026-04192-x (PMC13359804; doi:10.1186/s13059-026-04192-x)
Supplement: Supplementary file 1 — Figure S1. Release of transcriptional silencing in three new pol2a mutants. Figure S2. Contribution of H3K27me3 in POL2A-dependent gene silencing. Figure S3. POL2A is required for heterochromatin over-replication in atxr5/6. Figure S4. DNA repeats, H3K27me1 and H3K9me2 at pol2a-12 chromocenters. Figure S5. DNA methylation and H3K9me2 profiles in pol2a mutants. Figure S6. Comparison of pol2a and fas2 molecular phenotypes. Figure S7. Changes in small RNA accumulation in pol2a mutants. Figure S8. Characterization of pol2a cmt3 double mutants. Figure S9. DNA methylation profiles in mutant and drug contexts of replicative stress. Figure S10. Comparison of sequencing replicates. [file 13059_2026_4192_MOESM1_ESM.pdf]

Additional file 1

Table of contents

Figure S1. Release of transcriptional silencing in three new *pol2a* mutants.

Figure S2. Contribution of H3K27me3 in POL2A-dependent gene silencing.

Figure S3. POL2A is required for heterochromatin over-replication in *atxr5/6*.

Figure S4. DNA repeats, H3K27me1 and H3K9me2 at *pol2a-12* chromocenters.

Figure S5. DNA methylation and H3K9me2 profiles in *pol2a* mutants.

Figure S6. Comparison of *pol2a* and *fas2* molecular phenotypes.

Figure S7. Changes in small RNA accumulation in *pol2a* mutants.

Figure S8. Characterization of *pol2a cmt3* double mutants.

Figure S9. DNA methylation profiles in mutant and drug contexts of replicative stress.

Figure S10. Comparison of sequencing replicates.

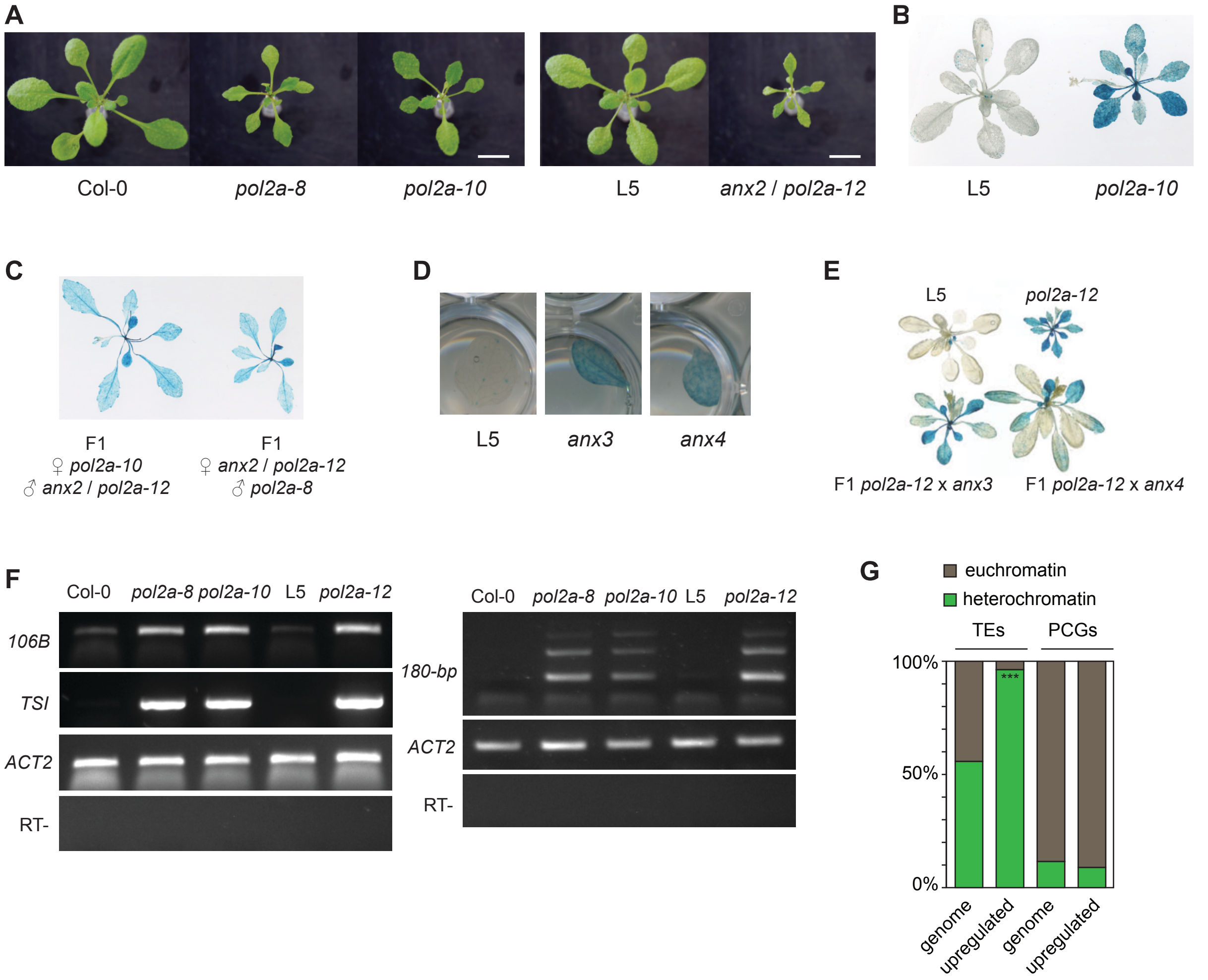

Figure S1. Release of transcriptional silencing in three new *pol2a* mutants

A Photos of 16-d-old L5 and *pol2a* mutant plants. Scale bar: 1cm. **B** L5-GUS transgene activity detected by X-Gluc histochemical staining in 3-week-old L5 and *pol2a-10* plants. **C-E** L5-GUS transgene activity detected by X-Gluc histochemical staining in crosses between *anx2* (*pol2a-12*) and *pol2a* mutant alleles (**C**), in *anx3* and *anx4* mutants (**D**) and in crosses between *pol2a-12*, *anx3* and *anx4* mutant alleles (**E**). The positions of punctual mutations are shown in figure 1C. **F** Transcript accumulation at endogenous repeats detected by RT-PCR. *ACTIN2* amplification is shown as a loading control. For each target, amplification in the absence of reverse transcription (RT-) was performed to control for genomic DNA contamination and one representative picture is shown. 180-bp repeats were amplified from RNAs that went through an extra round of DNase treatment, to ensure proper transcript quantification. The images are

representatives of three biological replicates. **G** Distribution of PCGs and TEs upregulated in *pol2a-12* between euchromatin and constitutive heterochromatin. Heterochromatin comprises pericentromeres and chromosome 4 heterochromatic knob according to Bernatavichute et al. (2008). Asterisks mark statistically significant deviations from the genomic distribution (*** : Z = -6.86, $P < 10^{-5}$; ns : not significant ($P > 0.05$)).

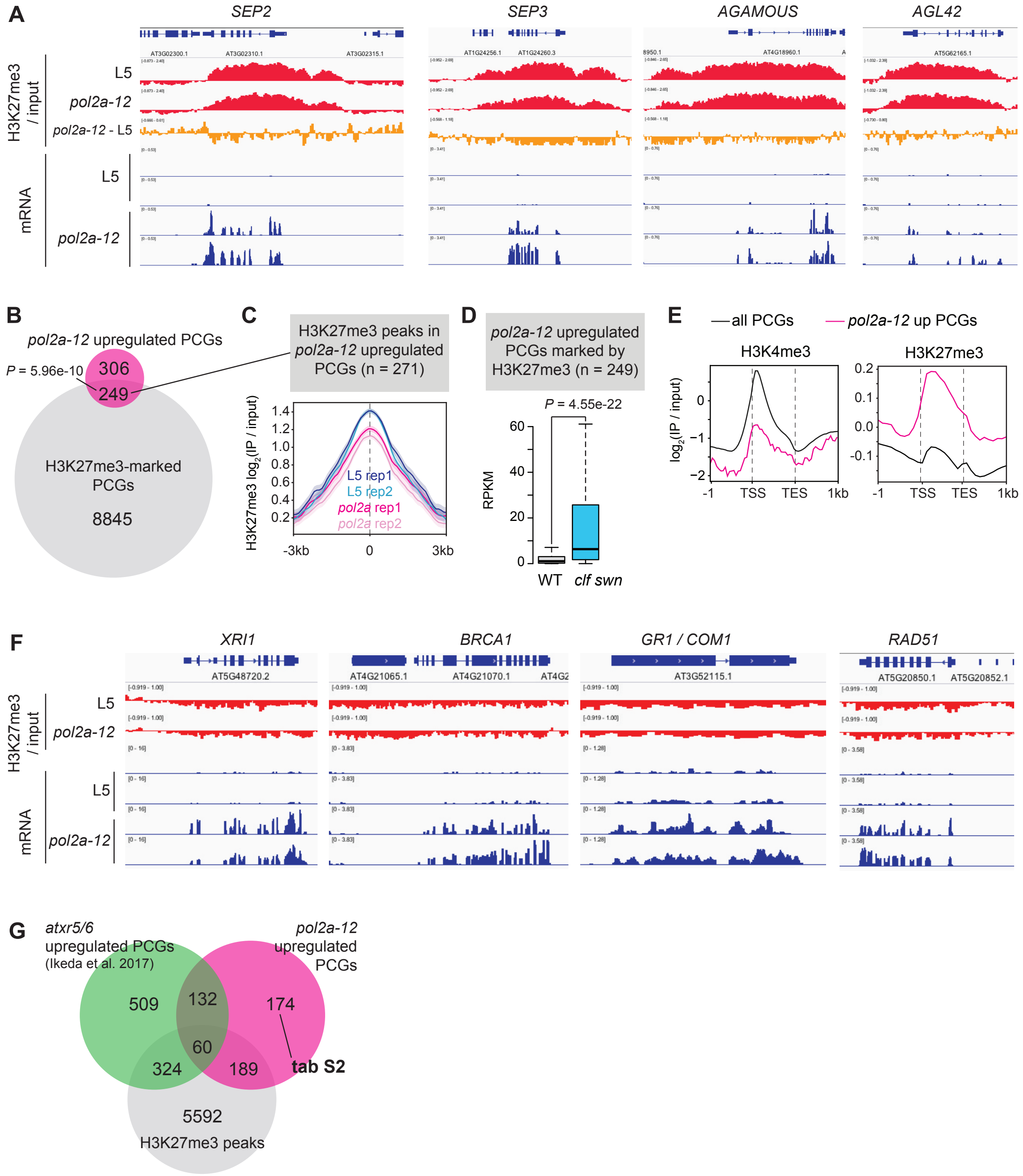

Figure S2. Contribution of H3K27me3 in POL2A-dependent gene silencing

A H3K27me3 enrichment (red, log_2 signal over input) and mRNA profiles (blue, RPM) at *SEP2*, *SEP3*, *AGAMOUS* and *AGL42* in L5 and *pol2a-12*. Two replicates are shown for mRNA-seq data. **B** Venn diagrams showing the proportion of PCGs upregulated in *pol2a-12* that are marked by at least one H3K27me3 peak. PCG coordinates were extended one kilobase upstream. The proportion of genes marked by H3K27me3 among *pol2a-12* upregulated PCGs

is significantly higher than the proportion expected by chance (hypergeometric test, *P* = 5.96e-10). **C** Metaplots showing average H3K27me3 enrichment (log_2 signal over input) in L5 and *pol2a-12* at H3K27me3 peaks found in *pol2a-12* upregulated PCGs that are overlapped by at least one H3K27me3 peak. Shaded areas show standard deviation. Two replicates are shown. **D** Transcript accumulation in reads per kilobase per million mapped reads (RPKM) at H3K27me3-marked PCGs that are upregulated in *pol2a-12*, in WT plants and *curly leaf swinger* (*clf swn*) double mutants (raw data from 39). The *P*-value from a two-sided unpaired Wilcoxon-Mann-Whitney test is indicated. **E** Metaplots of normalized ChIP-seq signal (log_2 signal over input) for H3K27me3 (this study) and H3K4me3 (GSM4275144) in WT plants over *pol2a-12* upregulated PCGs and all PCGs. **F** H3K27me3 enrichment and mRNA profiles at *RAD51*, *BRCA1*, *GR1/COM1* and *XRI1* in L5 and *pol2a-12* shown as in A. **G** Venn diagrams showing the overlap between PCGs upregulated in *pol2a-12* and *atxr5/6* (data from Ikeda et al. 2017), also showing the proportion of these genes that are overlapped by at least one H3K27me3 peak. PCG coordinates were extended one kilobase upstream.

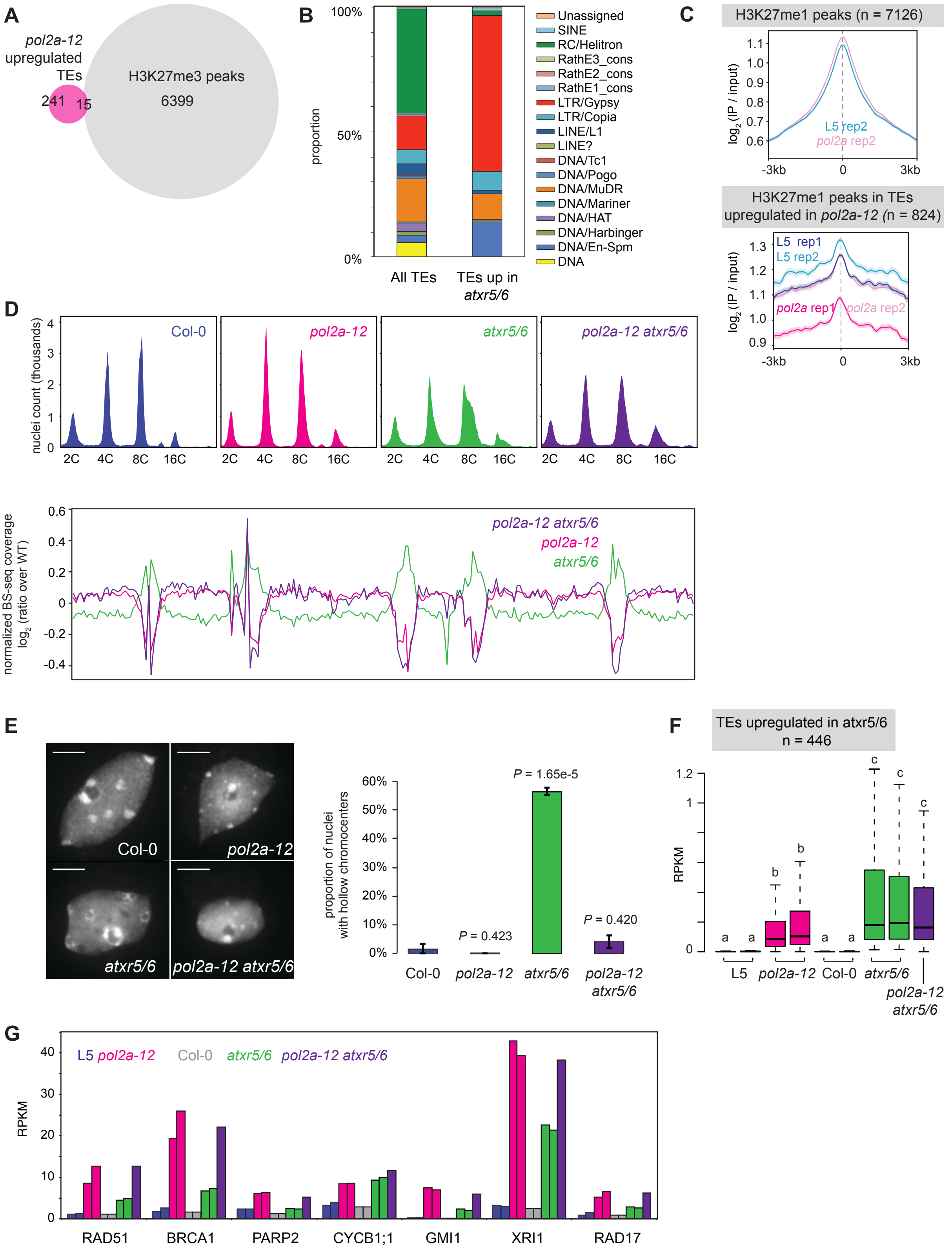

Figure S3. POL2A is required for heterochromatin over-replication in *atxr5/6*

A Venn diagrams showing the proportion of TEs upregulated in *pol2a-12* that are overlapped by at least one H3K27me3 peak. TE annotation coordinates were extended one kilobase

upstream. **B** Proportion of TE superfamilies among TEs upregulated in *atxr5/6* (data from Ikeda et al. 2017), with proportion from all TEs shown for comparison. **C** Metaplots showing H3K27me1 enrichment (log_2 signal over input) in L5 and *pol2a-12* at all H3K27me1 peaks (top) or at peaks found in *pol2a-12* upregulated TEs (bottom). Up to two replicates are shown. Shaded areas show standard deviation. TE coordinates were extended one kilobase upstream. **D** (top) Flow cytometry profiles generated with DAPI-stained nuclei extracted from rosette leaves of the indicated genotypes. Ploidy levels are indicated below peaks. (bottom) Log_2 ratios of BS-seq coverage in reads per million (RPM) in the indicated mutants normalized to their corresponding WT sample. Mean coverage was computed over non-overlapping 500 kb bins along the five *Arabidopsis* chromosomes. **E** (left) Representative DAPI-stained nuclei extracted from rosette leaves of the indicated genotypes. Scale bar: 5 µm. (right) Average proportion of nuclei with hollow chromocenters. Error bars represent standard error of the mean across three biological replicates. The *P*-value from an unpaired two-sided Student's t-test comparing each mutant with the WT is indicated. Heighty four to heighty height nuclei were analyzed for each replicate. **F** Transcript accumulation in reads per kilobase per million mapped reads (RPKM) at TEs upregulated in *atxr5/6* in indicated genotypes. The effect of genotype was verified with a Kruskal-Wallis rank sum test. Significant differences between groups were evaluated by a Dwass-Steel-Crichtlow-Fligner test and are indicated by lowercase letters ($P < 0.05$). Two biological replicates are shown for each genotype, except for *pol2a atxr5/6* where one sample was analyzed. **G** Transcript accumulation in reads per kilobase per million mapped reads (RPKM) at genes involved in the DNA damage response.

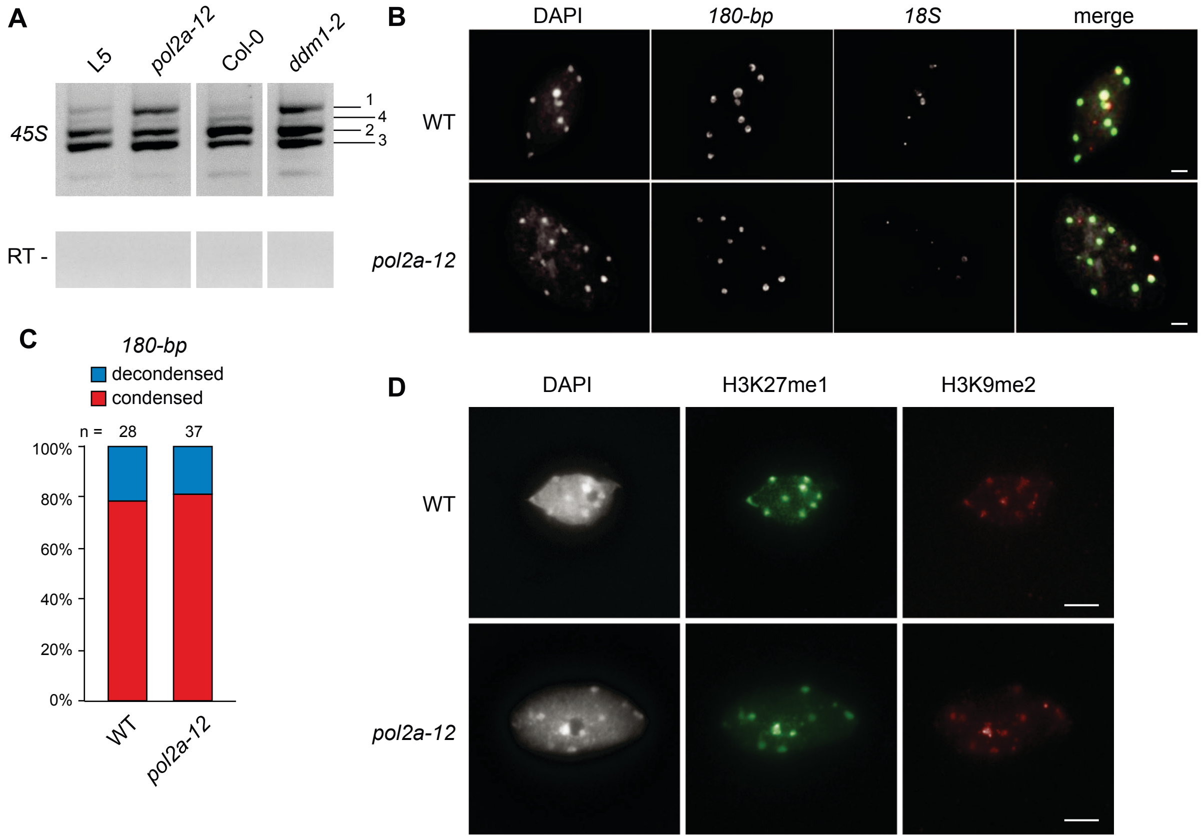

Figure S4. DNA repeats, H3K27me1 and H3K9me2 at *pol2a-12* chromocenters

A RT-PCR analysis of the 3' transcribed region of *45S* rDNA repeats on flowers of the indicated genotypes. Variants are indicated next to the gel. A PCR amplification without reverse transcription (RT-) was performed to control for genomic DNA contamination. Data is representative of three biological replicates. **B** Representative images showing DAPI-stained interphase nuclei from WT and *pol2a-12* hybridized with a probe for *180-bp* and *18S* rDNA repeats. Scale bar: 5 µm. **C** Proportion of nuclei identified as condensed or decondensed in WT and *pol2a-12*. No significant difference between the two genotypes was detected using a two-proportions Z-test ($P > 0.05$). **D** Distribution of H3K27me1 and H3K9me2 detected by immunofluorescence in interphase nuclei from WT and *pol2a-12* rosette leaves. Scale bar: 5 µm.

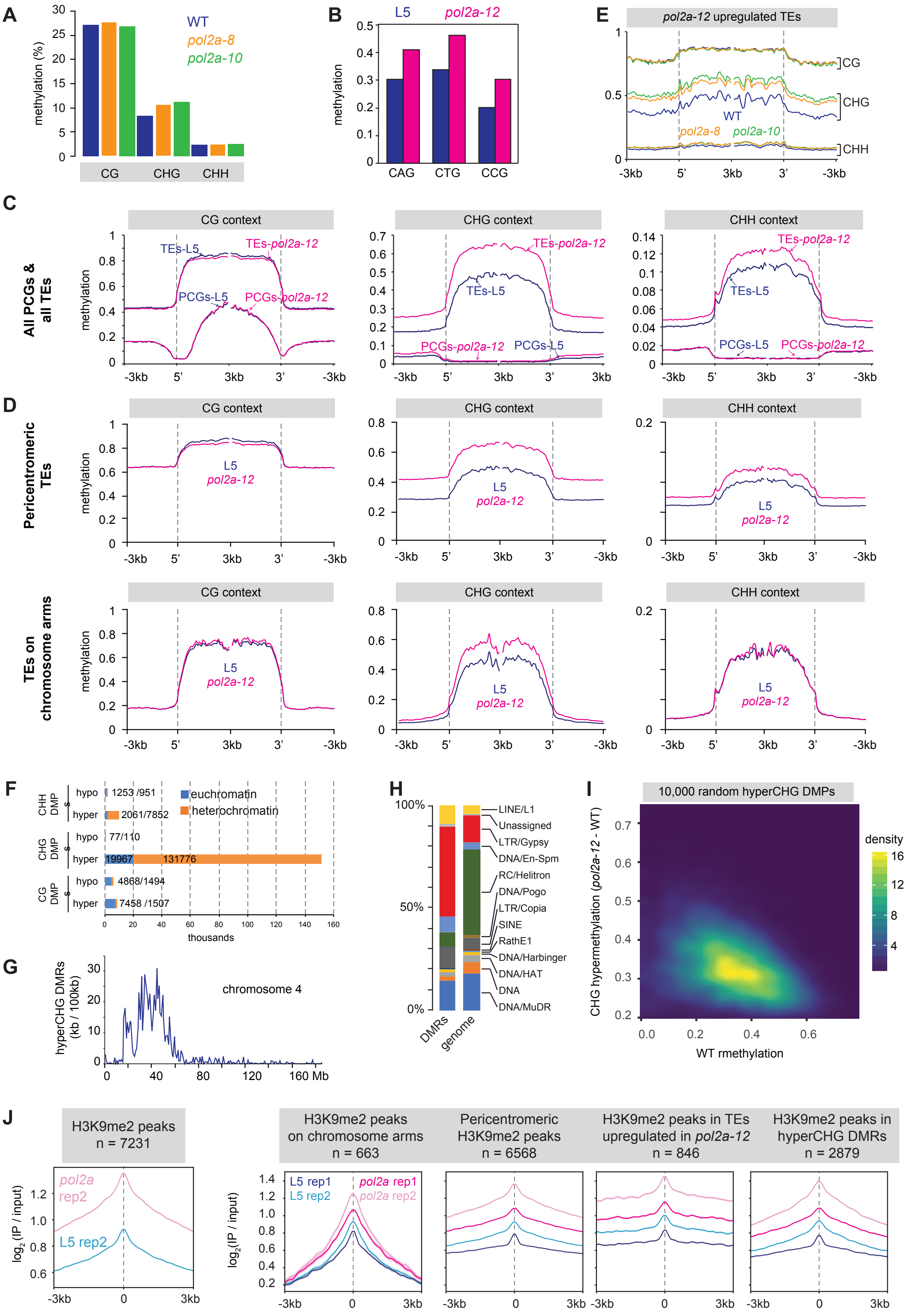

Figure S5. DNA methylation and H3K9me2 profiles in *pol2a* mutants

A Average methylation rates in CG, CHG and CHH contexts in Col-0 and *pol2a* mutants. **B** Average methylation levels in CHG subcontexts in L5 and *pol2a-12*. For each subcontext, positions unmethylated in both the WT and mutant samples were excluded. **C-D** Metaplots showing methylation rates in CG, CHG and CHH contexts in L5 and *pol2a-12* at TEs and PCGs. Annotations were aligned to their 5' or 3' end and average methylation was calculated for each 100-bp bin from 3 kb upstream to 3 kb downstream. In D, heterochromatic and euchromatic TEs were separated based on their genomic location (see methods). **E** Metaplots showing methylation levels at TEs upregulated in *pol2a-12*. **F** Differentially methylated positions (DMPs) identified in *pol2a-12* (see methods). DMPs were further sorted between euchromatin and heterochromatin based on their genomic location. **G** Distribution of *pol2a-12* hyper-CHG DMRs per consecutive non-overlapping 100 kb bins of chromosome 4, showing high proportion in heterochromatic regions. **H** Proportion of TE superfamilies among TEs intersected by *pol2a-12* hyper-CHG DMRs, with proportion for all TEs shown for comparison. **I** CHG hypermethylation in *pol2a-12* plotted against WT methylation levels shown for 10,000 random hyper-CHG DMPs. **J** Metaplots showing average H3K9me2 enrichment (log_2 signal over input) in L5 and *pol2a-12* at all H3K9me2 peaks (left) or at peaks located either in euchromatin, heterochromatin, intersecting *pol2a-12* upregulated TEs or *pol2a-12* hyper-CHG DMRs. Shaded areas show standard deviation.

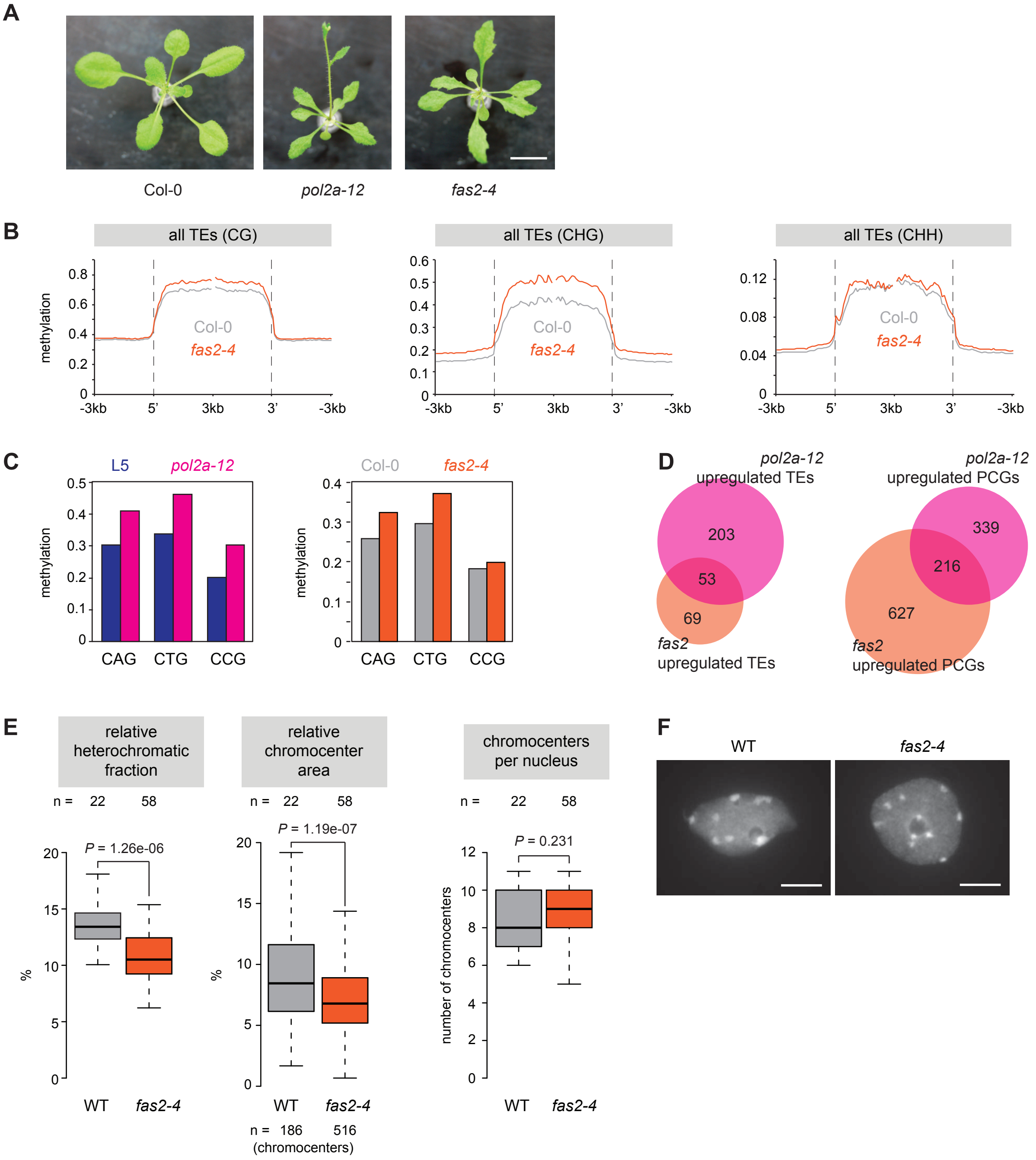

Figure S6. Comparison of *pol2a* and *fas2* molecular phenotypes

A Photos of 21-d-old plants. Scale bar: 1cm. Note that the Col-0 and *pol2a-12* images are the same individuals shown in Fig. S8D, as these plants were grown side-by-side and served as common controls for both experiments. **B** Metaplots showing methylation rates in CG, CHG and CHH contexts in *fas2-4* mutants at all TEs (data from Mozgova et al. 2018). Annotations were aligned to their 5' or 3' end and average methylation was calculated for each 100-bp bin from 3 kb upstream to 3 kb downstream. **C** Average methylation levels in CHG subcontexts in the

indicated samples, excluding positions unmethylated in both the WT and mutant samples in each comparison. **D** Venn diagrams showing the overlap between TEs and PCGs upregulated in *pol2a-12* and *fas2-4*. **E** Relative heterochromatic fraction (left), area of chromocenters normalized to the entire nucleus area (middle) and number of chromocenters per nucleus (right) quantified on DAPI-stained nuclei in WT and *fas2-4*. The number of nuclei analyzed is indicated on top. P-values from an unpaired two-sided Student's t-test are indicated. **F** DAPI-stained nuclei extracted from WT and *fas2-4* plants. Scale bar: 5 µm.

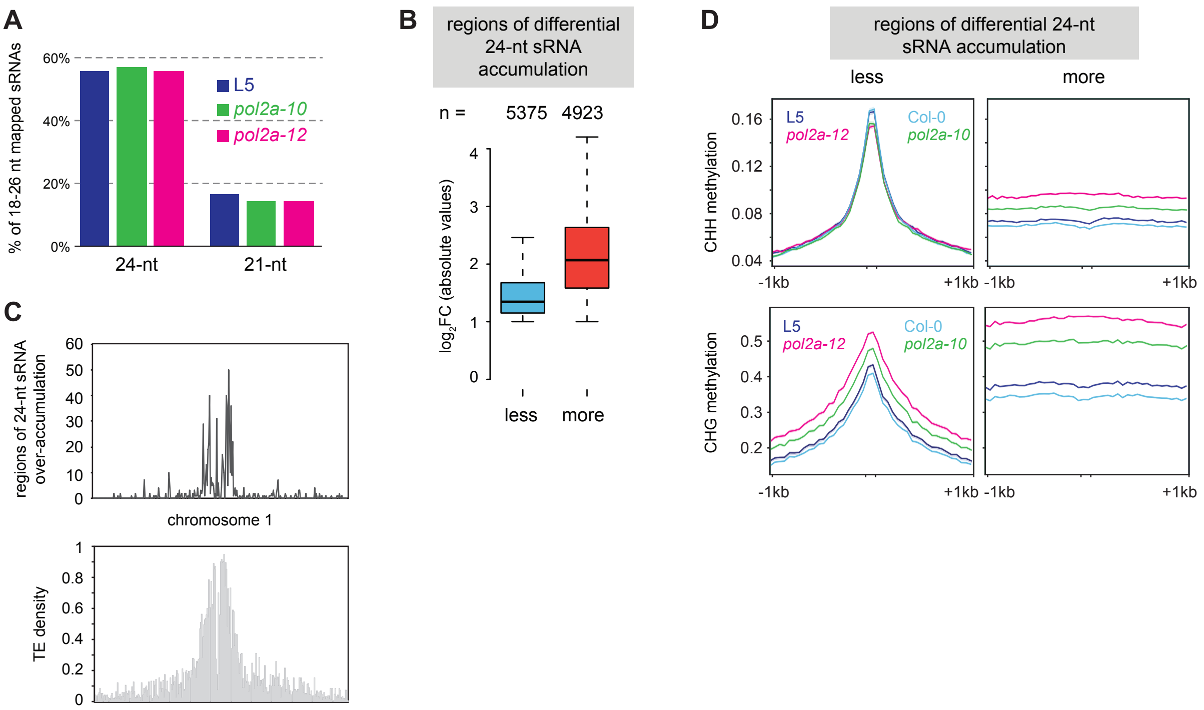

Figure S7. Changes in small RNA accumulation in *pol2a* mutants

A Overall proportion of 24-nt and 21-nt small RNAs (sRNAs) in L5, *pol2a-10* and *pol2a-12* flowers relative to the total number of mapped 18-26-nt sRNAs for each genotype. **B** Changes in 24-nt sRNA at 100-bp bins with significantly more or less 24-nt sRNAs represented as $\log_2$ fold change ($\log_2$FC) absolute values relative to L5. The number of regions is shown (top). **C** Number of 100-bp bins associated with 24-nt sRNA over-accumulation in *pol2a-12*, calculated per consecutive non-overlapping 100 kb bins of chromosome 1. TE density (bottom) is the proportion of TE annotations per 100 kb bins, showing the pericentromeric region. **D** Metaplots showing average DNA methylation rates in CHH (top) and CHG (bottom) contexts at 100-bp bins that gain or lose 24-nt sRNAs in *pol2a-12*, calculated for each bin from 1 kb upstream to 1 kb downstream.

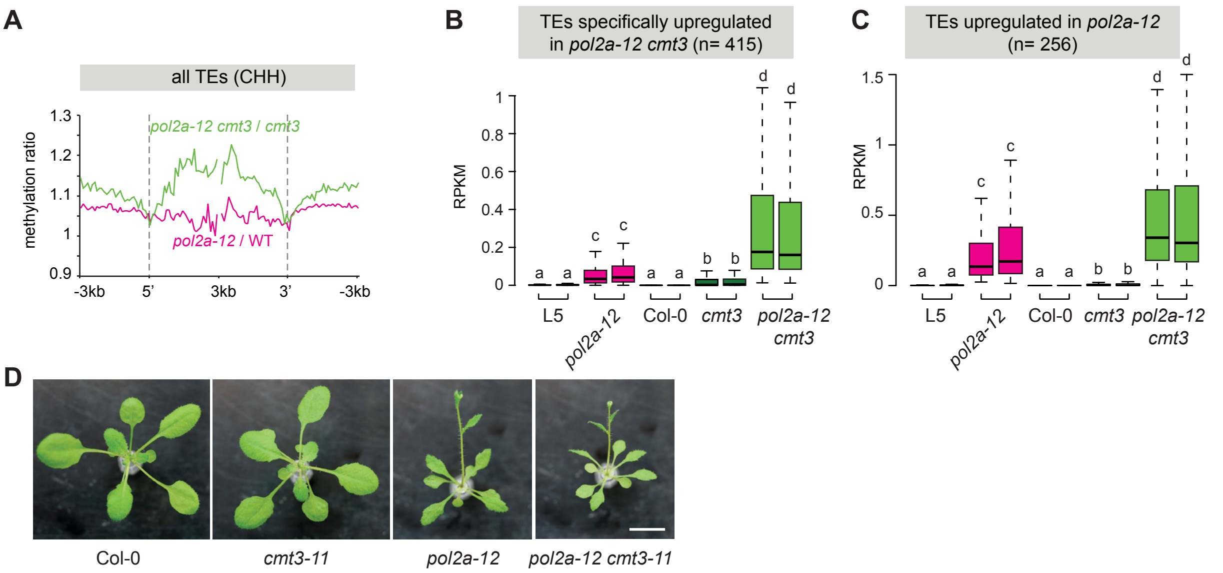

Figure S8. Characterization of *pol2a cmt3* double mutants

A TE methylation changes in CHH context in *pol2a-12* and *pol2a cmt3* normalized to WT and *cmt3*, respectively. Annotations were aligned to their 5' or 3' end and average methylation was calculated for each 100-bp bin from 3 kb upstream to 3 kb downstream. **B-C** Transcript accumulation in reads per kilobase per million mapped reads (RPKM) in indicated genotypes. The effect of genotype was verified with a Kruskal-Wallis rank sum test. Significant differences between groups were evaluated by a Dwass-Steel-Crichtlow-Fligner test and are indicated by lowercase letters (P < 5e-10 in B, P < 5e-2 in C). **D** Representative pictures showing 21-day-old plantlets of the indicated genotypes. Scale bar: 1cm. Note that the Col-0 and *pol2a-12* images are the same individuals shown in Fig. S8D, as these plants were grown side-by-side and served as common controls for both experiments.

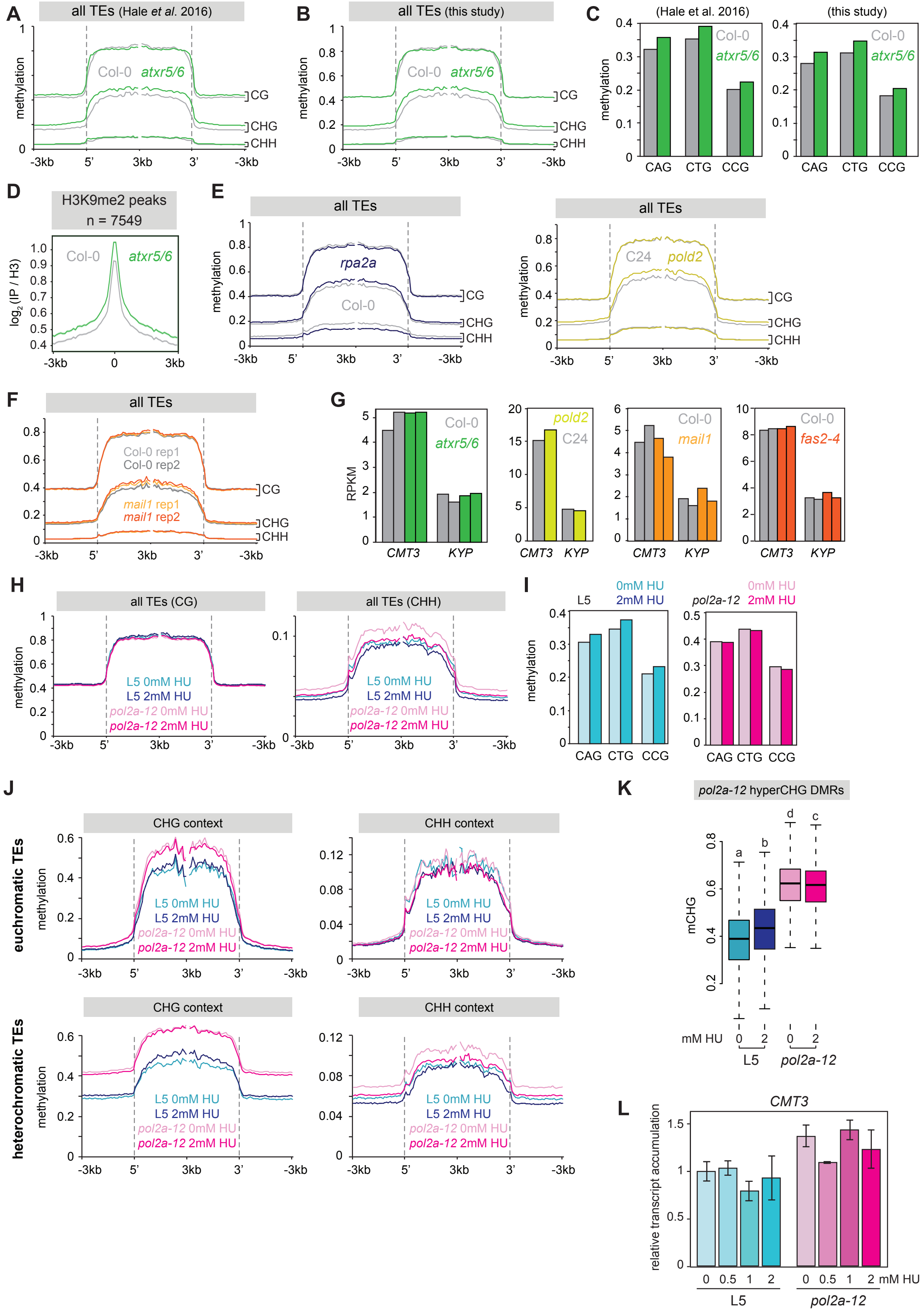

Figure S9. DNA methylation profiles in mutant and drug contexts of replicative stress

A-B Metaplots showing methylation rates in different contexts in *atxr5/6* at all TEs. Annotations were aligned to their 5' or 3' end and average methylation was calculated for each 100-bp bin from 3 kb upstream to 3 kb downstream. **C** Average methylation levels in CHG subcontexts in the indicated samples, excluding positions unmethylated in both the WT and mutant samples in each comparison. **D** Metaplots showing H3K9me2 enrichment (log_2 signal over H3) in WT and *atxr5/6* at H3K9me2 peaks. Shaded areas show standard deviation. Data from Ma et al. (2018). **E-F** Metaplots showing TE methylation in (**E**) *rpa2a* (Stroud et al. 2013), *pold2* (Zhang et al. 2016) and (**F**) *mail1* (Ikeda et al. 2017), represented as in A. **G** Transcript accumulation in reads per kilobase per million mapped reads (RPKM) for *CMT3* and *KYP* in *atxr5/6* and *mail1* (Ikeda et al. 2017), *pold2* (Zhang et al. 2016) and *fas2-4* (this study). **H** Metaplots showing TE methylation in L5 and *pol2a-12* treated or not with hydroxyurea (HU), represented as in A. **I** Average methylation levels in CHG subcontexts in the indicated samples, as in C. **J** Metaplots showing CHG and CHH methylation in the indicated samples at heterochromatic or euchromatic TEs (based on chromosomal location). **K** CHG methylation rates at *pol2a-12* hyperCHG DMRs in the indicated samples. We used a Kruskal-Wallis rank sum test followed by a Dwass-Steel-Crichtlow-Fligner test. Differences between groups are indicated by lowercase letters ($P < 4e\text{-}06$). **L** Transcript accumulation at *CMT3* analyzed by RT-qPCR in L5 and *pol2a-12* seedlings treated with various concentrations of HU, normalized to the *ACTIN2* gene with L5 0mM HU set to 1. No statistically significant differences were detected (two-sided unpaired Student's t-test, $P > 0.05$). Error bars represent standard error of the mean across three biological replicates.

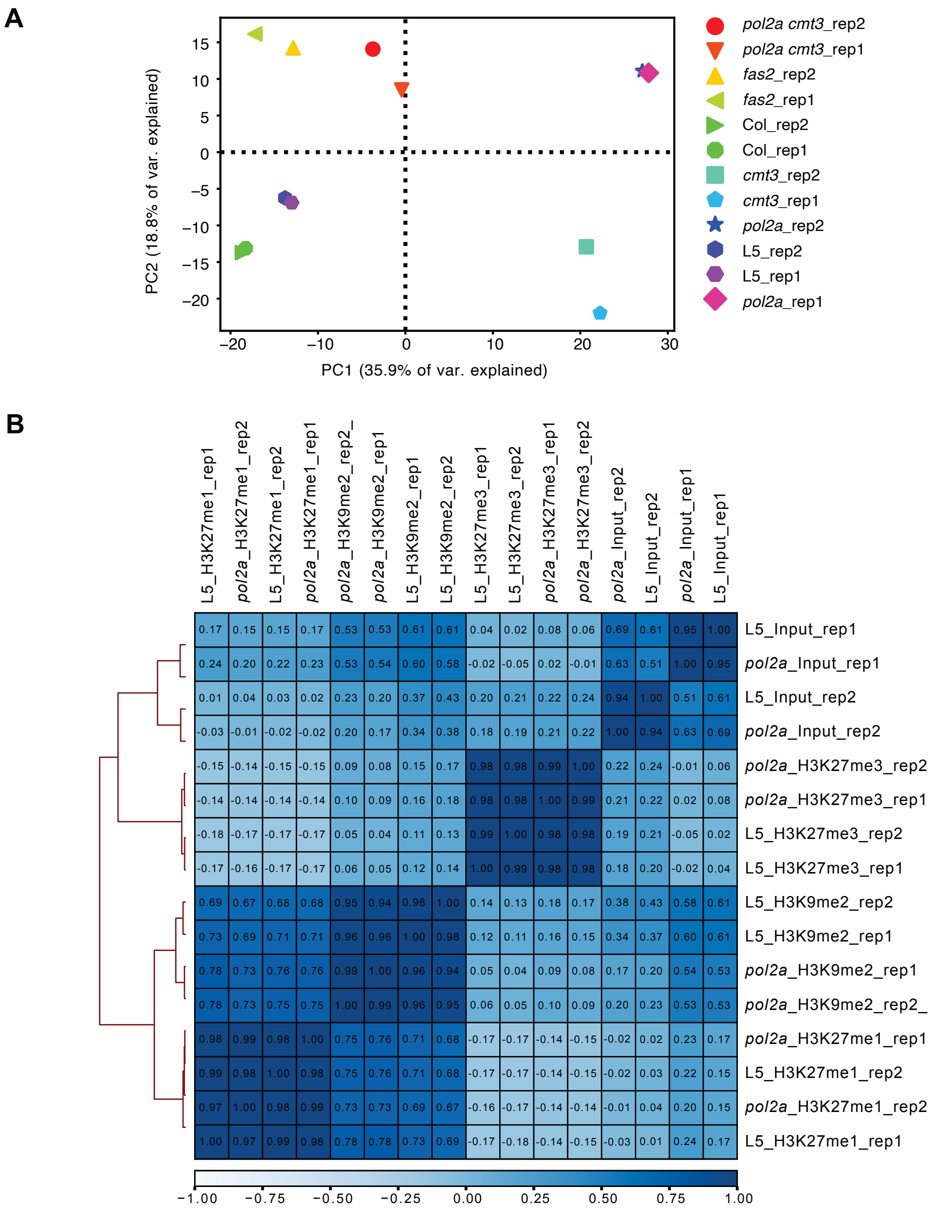

Figure S10. Comparison of sequencing replicates

A. Principal component analysis of normalized gene read counts in RNA-seq replicates used in this study. **B.** Hierarchical heatmap of ChIP-seq replicates based on Pearson correlation coefficients. Colors represent the correlation coefficients that are also indicated in each box. Clusters were constructed using complete linkage.
